# Malpighian tubules of *Rhodnius prolixus*: More than post-prandial diuresis

**DOI:** 10.3389/finsc.2023.1167889

**Published:** 2023-03-30

**Authors:** Ian Orchard, Areej N. Al-Dailami, Jimena Leyria, Angela B. Lange

**Affiliations:** Department of Biology, University of Toronto Mississauga, Mississauga, ON, Canada

**Keywords:** insect, transcriptome, immune response, antimicrobial peptides, detoxification, hormonal control, circadian rhythm, energy management

## Abstract

*Rhodnius prolixus*, a major vector of Chagas disease, may be considered the model upon which the foundations of insect physiology and biochemistry were built. It is an obligate blood feeder in which the blood meal triggers growth, development and reproduction. The blood meal also triggers a post-prandial diuresis to maintain osmotic homeostasis. In *R. prolixus*, as with other insects, the Malpighian tubules play a critical role in this diuresis, and much has been learned about diuresis in *R. prolixus*, and in other model insects. But the post-genomic era has brought new insights, identifying functions quite apart from diuresis for Malpighian tubules. Indeed, microarrays, transcriptomes, and proteomics have revealed the major roles that Malpighian tubules play in immunity, detoxification, pesticide resistance, and in tolerance to overall stress. This is particularly relevant to *R. prolixus* since gorging on blood creates several challenges in addition to osmotic balance. Xenobiotics may be present in the blood or toxins may be produced by metabolism of blood; and these must be neutralized and excreted. These processes have not been well described at the molecular level for Malpighian tubules of *R. prolixus.* This paper will review the involvement of Malpighian tubules in immunity and detoxification, identifying new aspects for Malpighian tubule physiology of *R. prolixus* by virtue of a transcriptome analysis. The transcriptome analysis indicates the potential of Malpighian tubules of *R. prolixus* to mount a robust innate immune response, and to contribute to antioxidant production and heme detoxification.

## 1 Introduction

All instars and adults of the kissing bug *Rhodnius prolixus* are obligate blood feeders and major vectors of Chagas disease in Central and South America. They are in fact blood gorgers and in the case of fifth instars can consume up to 10 times their unfed body weight in a single 20-minute feed. The blood meal triggers a variety of long term physiological and endocrinological events such as growth, development, metamorphosis, and reproduction (see 1, 2). The ability to trigger and therefore precisely time these events was taken advantage of by Sir Vincent Wigglesworth (e.g. 3, 4) and *R. prolixus* became a model insect upon which the foundations of insect physiology and biochemistry were built (see 5, 6).

The blood meal also triggers a critically important post-prandial diuresis, which is initiated within minutes of the start of gorging to lower the mass and concentrate the nutrients of the meal, improve mobility, and help avoid predation (see 1). Thus, *R. prolixus* rapidly excretes a fluid that is high in NaCl content and hypo-osmotic to the hemolymph, thereby eliminating 50% of the volume of the blood meal within 3 hours of gorging. Blood feeding creates several other challenges for the maintenance of homeostasis (see 7). Xenobiotics (pathogens and toxins) may be present in the blood or toxins may be produced by metabolism; and these also must be neutralized and excreted. Thus, in the longer term, *R. prolixus* must manage pathogens, an excess of K^+^ and Ca^2+^ from the digestion of blood cells, uric acid from nitrogenous waste, and organic anions from catabolism of blood proteins. In addition, hemoglobin is broken down, ultimately producing reactive oxygen species (ROS) that can cause tissue damage.

In *R. prolixus*, as with other insects, the Malpighian tubules play a critical role in diuresis, and again, *R. prolixus* became a model insect for the study of diuresis in insects through the pioneering work of a student of Wigglesworth, Simon Maddrell, and his colleagues (see 8–12). Insect Malpighian tubules are not innervated, and their fine control comes under the influence of the neuroendocrine system that releases amines and neuropeptides as diuretic or antidiuretic hormones. Autocrine factors can also be involved (13). These hormones regulate fluid secretion and reabsorption by the Malpighian tubules *via* a variety of receptors (G protein-coupled, tyrosine kinase, and guanylyl cyclase type receptors) linked to several second messenger systems that ultimately influence a V-type H^+^ ATPase, ion transporters and aquaporins, among others (see 1, 14–17). Much has been discovered about the control of diuresis in *R. prolixus*, and other model insects, but the post-genomic era has brought new insights, enabling the identification of novel diuretic and antidiuretic hormone-signaling pathways whilst also validating many of the existing models (see 1, 16–21). This era has also identified functions quite apart from diuresis for Malpighian tubules, and microarray and transcriptome analyses have revealed the major roles that Malpighian tubules play in immunity, detoxification, pesticide resistance, and in tolerance to overall stress (7, 16, 18, 20–22). Thus, Malpighian tubules from some insects have been shown to express immune-related genes responding to pathogenic challenge (e.g., bacteria, fungi, viruses and protozoa) by producing antimicrobial peptides (AMPs) such as attacin, defensin and cecropin. They also express organic solute transporters, detoxification-related genes such as cytochrome P450 monooxygenases (CYP450s), glutathione S-transferases (GSTs), carboxylesterases (CEs), and ATP-binding cassette (ABC) transporters; with phases I and II of detoxification capitalizing on the metabolic enzymes, and phase III of detoxification involving the transporters. Hence, Malpighian tubules may now be considered an immune responsive tissue, in addition to the canonical immune tissues, the fat body and the midgut (23–25).

Fascinatingly though, Malpighian tubules of some insects have also evolved quite specialized functions (not covered in this review), whereby there have been modifications of entire tubules, specific segments, or specific secretory cells scattered along the tubules. Some examples are the production of mucopolysaccharides for nest building, production of adhesive secretions for the oothecae, and remarkably, the phenomenon of emitting bioluminescence from “light organs” developed from the swollen distal tips of the tubules for the attraction of prey and partners for mating (see 26).

With immunity in mind, it is worth noting that *R. prolixus*, like other Triatomines, can ingest *Trypanosoma cruzi* trypomastigotes present in the blood meal. In contrast to other vector borne parasites (such as *Trypanosoma rangeli* in *R. prolixus*) that invade the hemocoel and replicate in the salivary glands, *T. cruzi* replicates only within the digestive tract, from where *R. prolixus* can transmit the infective forms (present in the hindgut), in its urine and feces following gorging (24). If *T. cruzi* enters the hemocoel of *R. prolixus* it is killed by the innate immune system, but this does not occur for *T. rangeli*. In addition, though, an obligate symbiotic bacterium, *Rhodococcus rhodnii*, obtained when first instars eat feces of other *R. prolixus*, is found in the anterior midgut (27). There must therefore also be mechanisms in place to recognize beneficial members of the microbiota whilst eliminating pathogens, adding some complexity to blood feeding, immunity, and indeed vector control (see 24).

The genome of *R*. *prolixus* has been sequenced (28), providing an advantage for scientific advancement in the biology of *R. prolixus*. This review will focus on insights into new functions of *R. prolixus* Malpighian tubules brought about by mining transcriptomes of Malpighian tubules taken from unfed fifth instars, and from fifth instars 3 h and 24 h post blood meal (PBM). This analysis reveals the expression of transcripts that are unlikely to be involved in the elimination of excess water and salts *per se*, but in other, quite distinct physiological processes. It must be clearly noted though, that transcripts alone do not prove a process, and further research is required to understand the true physiological meaning of these putative non-diuretic actions. The transcript expression does, however, point in directions for further exploration.

## 2 Malpighian tubule transcriptome

In a previous work, we reported a transcriptome study of *R. prolixus* Malpighian tubules, conducted *via* RNA sequencing (RNA-seq) and reference genome-based transcriptome assembly (deposited under Bioproject PRJNA729781), mainly focusing on diuresis (1). *R. prolixus* has 4 Malpighian tubules, which, as in other insects, are blind-ended tubes that freely lie in the hemocoel, emptying their secretions into the gut at the midgut-hindgut junction. To perform a transcriptome analysis of Malpighian tubules before and after the metabolically demanding process of gorging and diuresis, Malpighian tubules were dissected from fifth instar *R. prolixus* at 15 d post-ecdysis (unfed condition) and 3 h and 24 h PBM. In the unfed condition, *R. prolixus* are in a state of arrested development and can go several months without a blood meal, but when they take a blood meal it triggers short term and long term physiological events as mentioned earlier (see 1). A bioinformatic pipeline to process RNA-seq reads for mapping sequences, transcript quantification, differential expression analysis and Kyoto encyclopedia of genes and genomes (KEGG) enrichment as well as the validation of RNA-seq data, have been thoroughly detailed by Orchard et al. (1) and Leyria et al. (29, 30). Here, we performed heatmap analysis to compare mRNA expression levels under the different feeding states presented by means of a color scale, showing the Fragments Per Kilobase Million (FPKM) values obtained by gene expression analysis after normalization. All the numeric information of the heatmap charts is shown in several worksheet tabs in **Supplementary Worksheet 1** (3 h PBM vs. unfed) and **Supplementary Worksheet 2** (24 h PBM vs. unfed).

**Figure 1:**
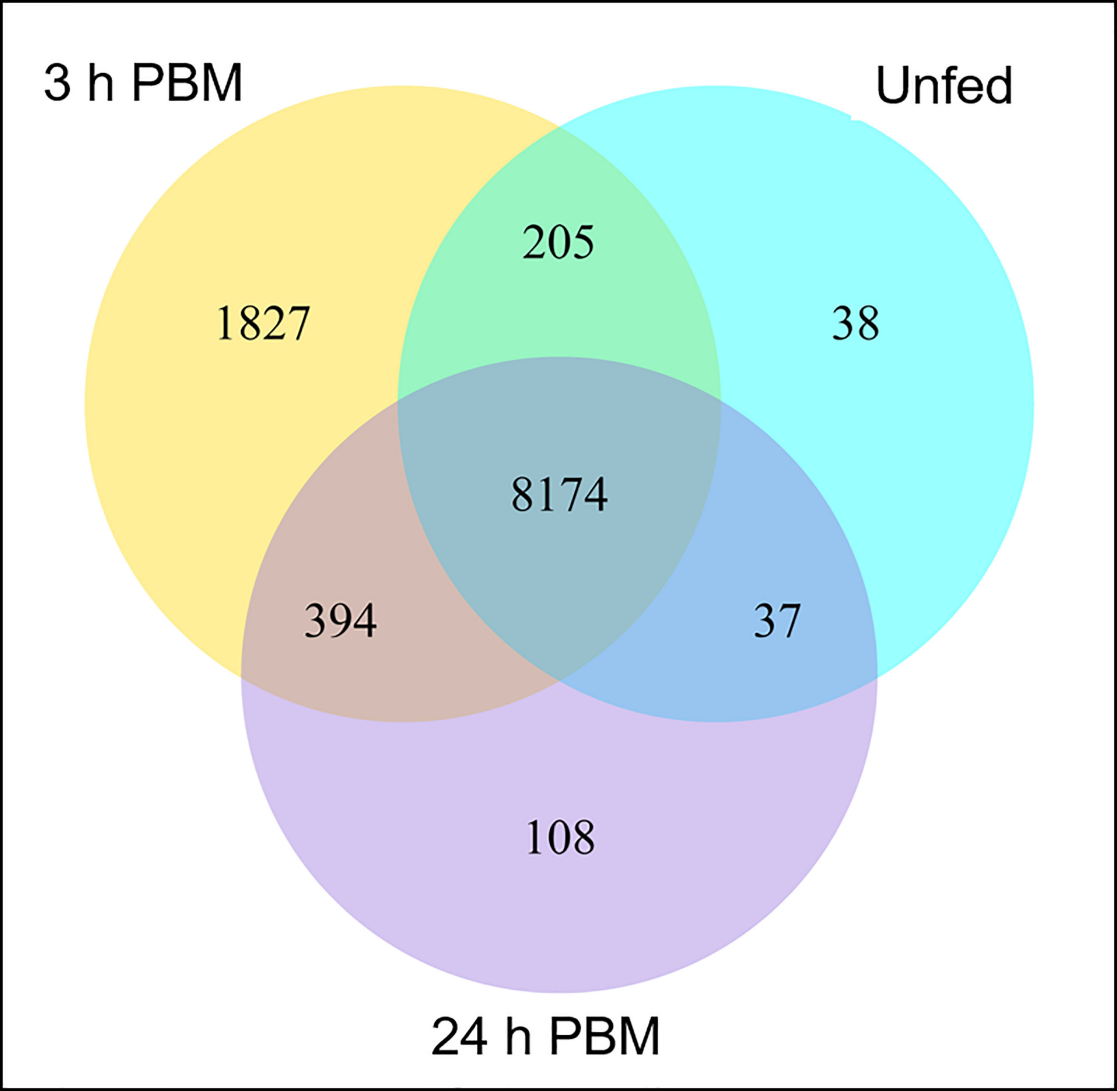
Gene co-expression. Venn diagram presents the number of genes that are uniquely expressed within each group (unfed, 3 h PBM and 24 h PBM), with the overlapping regions showing the number of genes that are expressed in the three groups. h PBM, hours post blood meal. Default threshold of FPKM value is set to 1.

**Figure 2:**
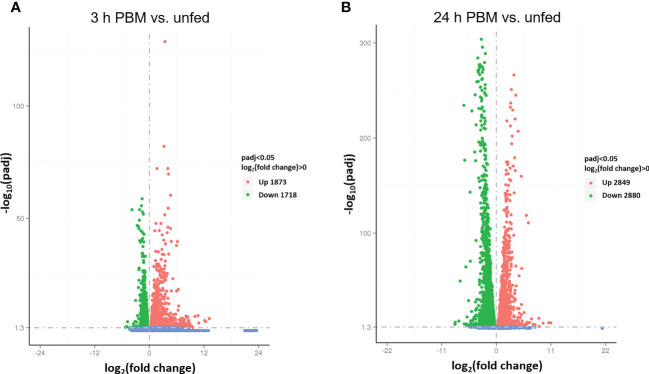
Screening of differentially expressed genes (DEG) by volcano plots. **(A, B)** The x-axis shows the fold change in gene expression log_2_(fold change) of 3 h PBM vs. unfed **(A)** or 24 h PBM vs. unfed **(B)** and the y-axis shows the statistical significance of the differences − log_10_(padj). Significantly up and down regulated genes are highlighted in red and green, respectively. Genes that did not express differently are showed in blue. Readcount values used to calculate *fold changes* are after normalization (obtained by DESeq); p-adj is the p-value after normalization (obtained by negative binomial distribution method). Threshold was set as padj < 0.05. h PBM, hours post blood meal.

Interestingly, 38, 1827, and 108 genes are uniquely expressed in the unfed condition, 3 h PBM, and 24 h PBM, respectively, whereas 8174 genes are commonly expressed in all nutritional states (**Figure 1**). It appears that at 3 h PBM, the expression of the unique genes has occurred, which coincides with an appropriate time point for the metabolically demanding diuresis. Of the overlapping genes in the 3 nutritional conditions, the transcriptome analysis shows a comparable number of the differentially expressed genes (DEGs) that are upregulated and downregulated between each fed and unfed condition (3 d PBM vs. unfed and 24 d PBM vs. unfed) (**Figure 2**). These support the idea that Malpighian tubules not only demand energy during the post-prandial diuresis but also in the unfed condition, presumably due to the activity of antidiuretic/conservation processes. Using cluster analysis, 4 major clusters that are regulated by nutritional state can be identified, illustrating gene expression patterns which may have similar functions or take part in the same biological process. When heatmaps of these clusters throughout the 3 experimental groups are analyzed, we can visually observe a nutrition-dependent pattern of physiological responses (**Supplementary Figure 1**). In the following sections we have performed an in-depth exploration of these DEG in Malpighian tubules, describing changes induced by blood gorging that are distinct from those associated with diuresis, which were previously covered in Orchard et al. (1).

## 3 Immunity

### 3.1 Immune pathways and AMPs

The arthropod immune system relies exclusively on an innate response triggered by invading organisms (see 24, 27, 31). The immune response is coordinated by the immune deficiency (IMD) pathway, the Toll pathway, the Janus kinase (JAK)-signal transducer and activator of transcription (STAT) pathway (JAK-STAT pathway), and the RNA interference (RNAi) pathway (see 27). While each of these pathways has typically been associated with subgroups of pathogens, there is cross talk between the various pathways including synergistic interactions (27, 32). Immune effector molecules include AMPs, nitric oxide, phenoloxidases, and protease inhibitors. Much of the information concerning these pathways has been obtained from studies in *Drosophila melanogaster* (see 33–35) but these pathways are now well described in *R. prolixus* for reviews (see 24, 27). Infections with trypanosomes and bacteria trigger a tissue specific immune response in *R. prolixus*, modulating the expression of AMPs in the fat body and/or midgut that include lysozymes A and B (Lys-A and B), prolixicin, and defensins A-C (36, 37).

Insect Malpighian tubules are essentially free floating in the hemolymph and are now recognized as immune sensing organs with an important role in innate immunity. They can sense a threat, and mount a defense by secreting AMPs, independent of the fat body, the primary immune organ (22, 38). For example, in *D. melanogaster*, Malpighian tubules express all the components of the IMD and Toll pathways and produce several AMPs in response to immune challenges (see 39). *AMP* transcripts are actually constitutively expressed in *D. melanogaster* tubules, and AMP production, which is upregulated several fold in response to an immune challenge, relies upon the IMD pathway and is independent of the Toll pathway (40). In *Aedes aegypti* Malpighian tubules, upregulation of the Toll pathway results in the expression of immune effectors, including *AMPs* (22), and in the leaf hopper *Psammotettix striatus*, *AMPs* are highly expressed, but differentially, in regions of the Malpighian tubules (41).

**Figure 3:**
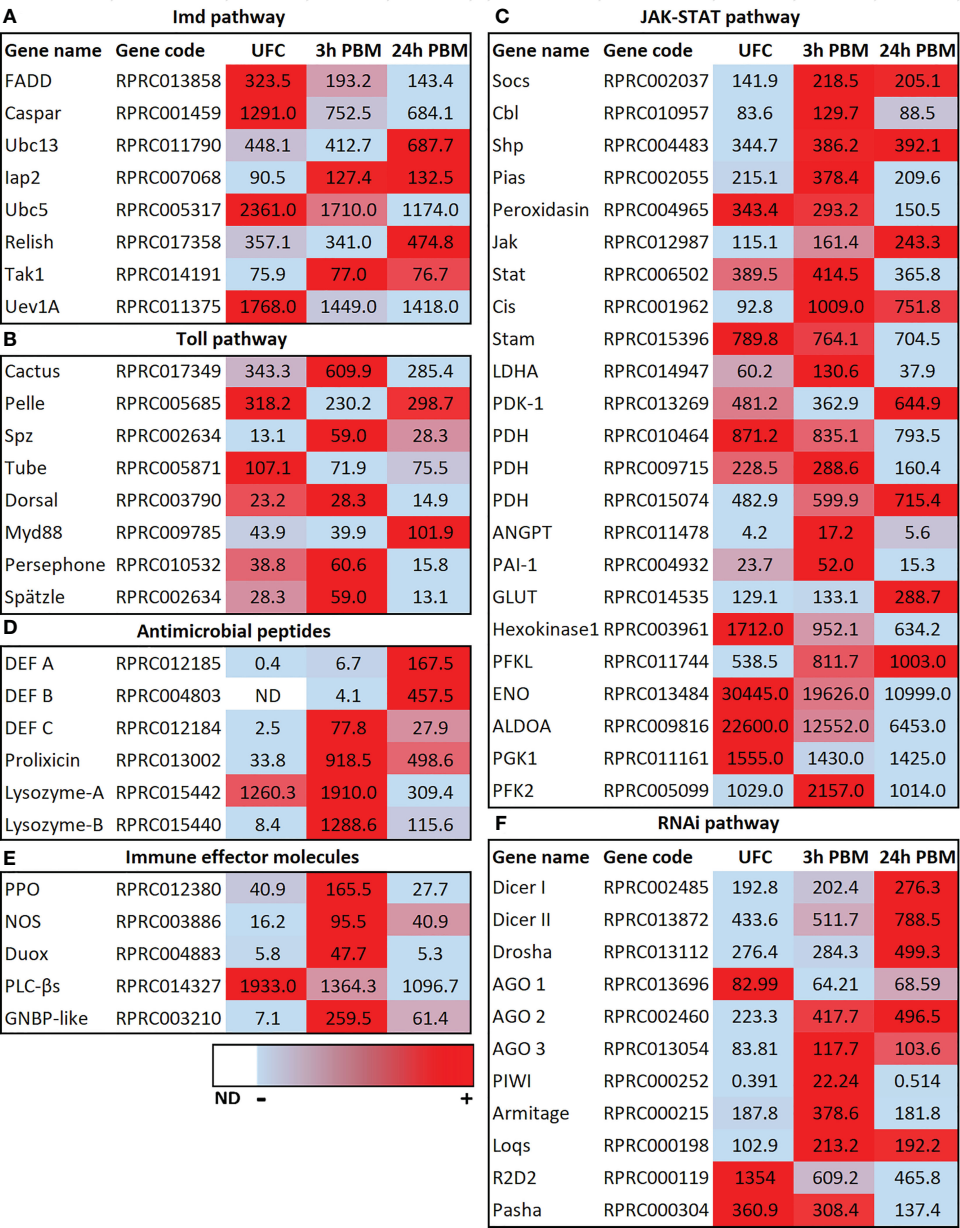
Heat map comparing the mRNA expression levels of **(A)** IMD, **(B)** TolL, **(C)** JAK-STAT, **(D)** antimicrobial peptides, **(E)** immune effector molecules and **(F)** RNAi signaling pathways in Malpighian tubules from insects in the unfed condition (UFC), 3 h and 24 h post blood meal (PBM). The input data is the readcount value from gene expression level analysis after normalization and is presented by means of a color scale, in which light blue/red represent lowest/highest expression and white indicate transcript expression is 0 or not detected (ND). DESeq was used to perform the analysis. Details of transcript expression are shown in **Supplementary Worksheets 1, 2**.

In *R. prolixus* the transcripts for the *IMD, Toll*, *JAK-STAT*, *AMPs*, and *RNAi* pathways are present in the Malpighian tubule transcriptome, suggesting an involvement of Malpighian tubules in *R. prolixus* innate immunity. For example, transcripts for these pathways are present in the Malpighian tubules of unfed *R. prolixus*, and some of them are modulated by blood gorging (**Figure 3**; **Supplementary Worksheets 1, 2**), further suggesting that blood gorging might trigger aspects of innate immunity. In particular, the transcript for *Relish*, a transcription factor in the IMD pathway which upon cleavage interacts with other proteins to modulate expression of *AMP* genes, is significantly upregulated 24 h PBM (**Figure 3A**). The transcript for *Caspar*, which inhibits one arm of the IMD pathway, is significantly downregulated at 3 h and 24 h PBM (**Figure 3A**). The Toll receptor is activated by a processed form of a ligand, Spätzle (Spz), and the transcript for *Spätzle* is significantly upregulated at 3 h but not 24 h PBM (**Figure 3B**). On the other hand, the transcript for *Cactus* which inhibits the Toll pathway is significantly upregulated at 3 h, but not at 24 h PBM, and the basal transcript level of the transcription factor *Dorsal* is not altered at 3 h or 24 h PBM (**Figure 3B**). Indeed, by KEGG pathways analysis, we can identify a number of DEGs enriched at 3 h PBM that belong to Toll and IMD signaling pathways (**Supplementary Figure 2**). The JAK-STAT pathway which was originally identified as a cytokine signaling pathway in mammals is now known to be an antiviral defense in *A. aegypti* and *D. melanogaster* (42). The transcript for the transcription factor *STAT* is not upregulated at 3 h or 24 h PBM, relative to its basal rate in unfed *R. pro*lixus, but transcripts for *Socs*, an inhibitor of the JAK-STAT pathway is significantly upregulated at 3 h and 24 h PBM (**Figure 3C**).

The changes seen PBM would appear to suggest the IMD pathway, in particular, might be regulating AMPs and so it is particularly interesting to note the upregulation of transcripts for *AMPs* (**Figure 3D**). Thus, the expression of transcripts for *AMPs* in Malpighian tubules from unfed insects is very low, but expression is induced by blood gorging, with *defensin C* and *prolixicin* significantly upregulated at 3 h and 24 h PBM, and transcripts for *defensins A* and *B* significantly upregulated at 24 h PBM (**Figure 3D**). Prolixicin was identified as a novel AMP isolated from *R. prolixus* with differential activity against bacteria and *T. cruzi* (43). Thus, recombinant prolixicin has strong activity against several gram positive and negative bacteria, but no significant toxicity against *T. cruzi*. The transcript for *prolixicin* is significantly and highly upregulated at 3 h PBM and 24 h PBM (**Figure 3D**).

Gram negative binding proteins (GNBPs) were first characterized in *Bombyx mori* that bind to *E. coli*, and subsequently several GNBPs were identified in insects to bind gram-negative bacteria (see 44). In *R. prolixus* Malpighian tubules, the transcript for *GNBP* (whose product recognizes proteins in the bacterial cell wall) is significantly upregulated at 3 h PBM and remains upregulated from the unfed basal level at 24 h PBM (**Figure 3E**; **Supplementary Worksheets 1, 2**).

Lysozymes also play an important role in innate immunity, providing protection mainly against bacteria, but also against viruses, and fungi. In *Manduca sexta*, the Malpighian tubules express the second highest levels of *lysozyme* transcript, behind fat body, and levels are increased following challenge with peptidoglycan (45). The *R. prolixus* Malpighian tubule transcriptome reveals 4 transcripts (*Lys-A* and *Lys-B*, and 2 fragments) for lysozymes. *RpLys-A* is mainly expressed in the midgut after ingestion of a blood meal containing *T. cruzi*, and *RpLys-B* is expressed primarily in the fat body after bacterial injection (46). *RpLys-A* and *B* are significantly upregulated at 3 h PBM, but their transcript levels then decrease from these levels at 24 h (**Figure 3D**).

### 3.2 Prophenoloxidase

Prophenoloxidase (PPO) is a humoral protein that can induce melanization around invading pathogens after activation and induce cellular and humoral immunity (35, 44). Intermediates produced in the melanization process can kill bacteria directly (47). Here we find that the *PPO* transcript is significantly upregulated 3 h PBM, but returns to basal levels 24 h PBM (**Figure 3E**). Nitric oxide synthase (NOS) catalyzes the production of nitric oxide which is an important signaling molecule that can act as a retrograde neurotransmitter but is also used as an immune defense mechanism. In *D. melanogaster*, nitric oxide and cGMP can activate the IMD pathway and *AMP* expression in a variety of tissues, including Malpighian tubules (see 39), and NOS is strongly upregulated in Malpighian tubules of *Anopheles gambiae* infected with *Plasmodium* (see 15). The transcript for *NOS* in *R. prolixus* Malpighian tubules is significantly upregulated 3 h and 24 h PBM, suggesting a possible involvement in the immune response (**Figure 3E**).

### 3.3 Phospholipase C-β

Phospholipase C-β (PLCβ) signaling modulates dual oxidase (DUOX) activity to produce microbicide ROS. These have been studied in the *D. melanogaster* digestive systems where contact with bacteria rapidly activates PLCβ to mobilize intracellular Ca^2+^ through inositol 1,4,5-trisphosphate for DUOX-dependent reactive oxygen species activation. This signaling pathway can act as a first line of defense but is also involved in discriminating between symbionts and pathogens (48). Interestingly, the transcript for *PLC*β is highly expressed in tubules from unfed *R. prolixus*, and slightly downregulated 3 h and further downregulated 24 h PBM (**Figure 3E**). The *DUOX* transcript though is low in unfed *R. prolixus*, but significantly upregulated 3 h PBM (**Figure 3E**).

### 3.4 RNAi pathway

In insects, as in other organisms, RNA interference (RNAi) mediates antiviral immunity (see 49). RNAi silences gene expression through small interfering RNAs (siRNAs) and microRNAs (miRNAs). In *D. melanogaster* Dicer-2 produces siRNAs, whereas Dicer-1 recognizes precursors of miRNAs. The small RNAs are assembled with an Argonaute protein into related effector complexes, such as RNA-induced silencing complex (RISC), to guide specific RNA silencing. Interestingly, in *R. prolixus* Malpighian tubules, transcripts in the RNAi pathway, *Dicer I, II* and *Drosha*, are significantly upregulated at 24 h PBM but not at 3 h PBM (**Figure 3F**), while the transcripts for double stranded RNA-binding motifs *R2D2* and *pasha* are significantly downregulated 24 h PBM (with *R2D2* significantly downregulated at 3 h). The *RISC* transcripts *Argonaut 2* and *3* are upregulated at 3 h PBM and *Argonaut 2* remains upregulated at 24 h PBM.

The above results imply a robust activation and regulation of the innate immune response by Malpighian tubules of *R. prolixus*, as shown for some other insects. We speculate that there may be synergistic effects among the immune pathways but further experimentation is required to confirm these observations.

## 4 Detoxification

**Figure 4:**
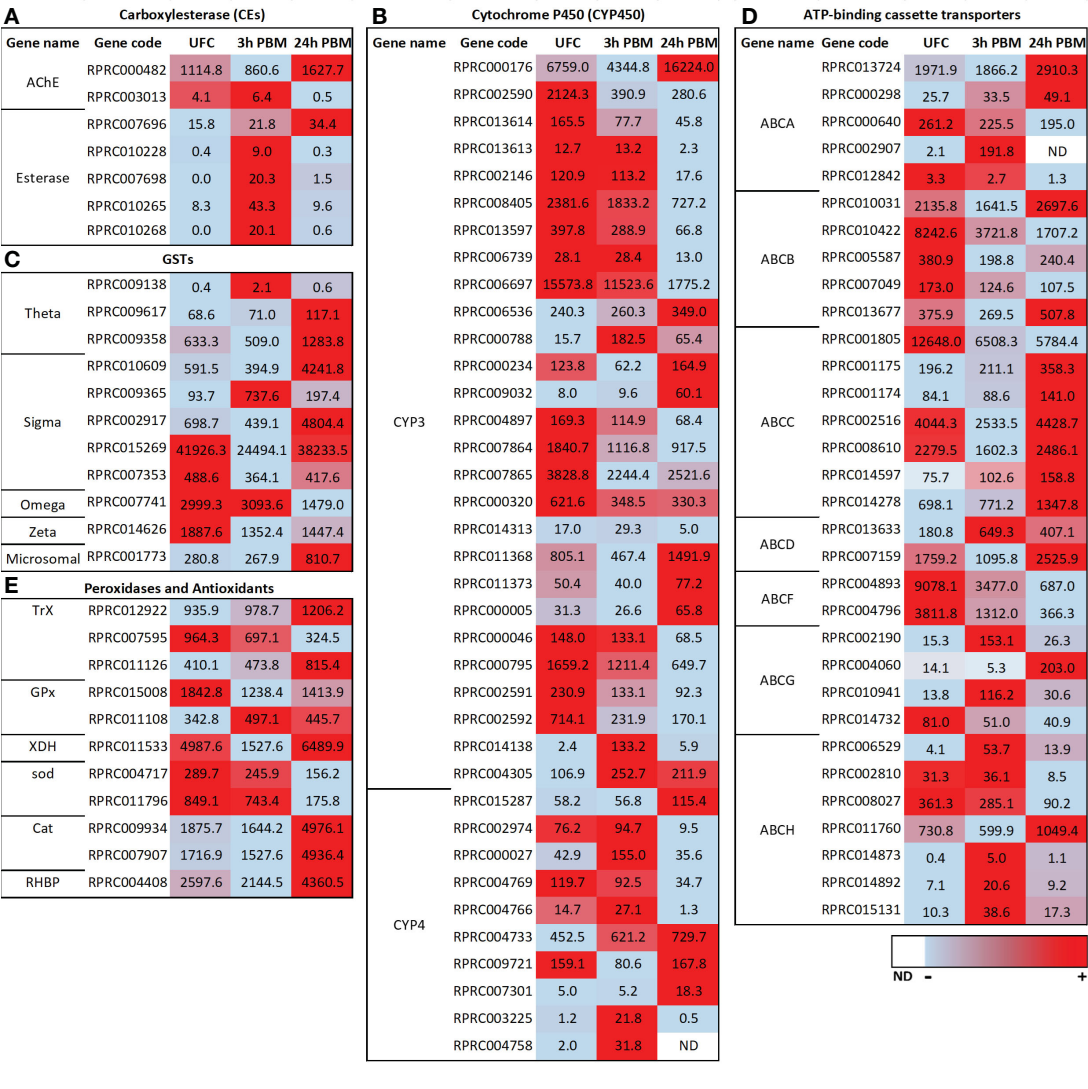
Heat map comparing the mRNA expression levels of **(A)** carboxylesterase (CEs), **(B)** cytochrome P450s (CYP3 and CYP4), **(C)** glutathione S-transferases (GSTs), **(D)** ATP-binding cassette (ABC) transporters and **(E)** peroxidases and antioxidants in Malpighian tubules from insects in the unfed condition (UFC), 3 h and 24 h post blood meal (PBM). The input data is the readcount value from gene expression level analysis after normalization and is presented by means of a color scale, in which light blue/red represent lowest/highest expression and white indicate transcript expression is 0 or not detected (ND). DESeq was used to perform the analysis. Details of transcript expression are shown in **Supplementary Worksheets 1, 2**.

As mentioned earlier, Malpighian tubules are active in the detoxification and excretion of xenobiotics and toxins, and resistance to pesticides (7, 15, 16, 20, 50). For example, there are several genes that are abundant or highly enriched in the Malpighian tubules of *D. melanogaster* which participate in metabolism and detoxification, including the genes that express alcohol dehydrogenase, glutathione transferase, and cytochrome P450s. Indeed, transcripts for several cytochrome P450s are heavily enriched in the tubules. Glutathione transferases play a role in phase II metabolism of xenobiotics, conjugating reduced glutathione to lipophilic substrates, making them more hydrophilic, and thus more easily excreted. Insecticide resistance has also been directly attributable to overexpression of a cytochrome P450 or glutathione-S-transferase, which influence survival upon DDT challenge in *D. melanogaster* (see 15). These findings suggest the possibility of the Malpighian tubules being an important site for detoxification and determining insecticide resistance. Furthermore, changes in transcript abundance in the Malpighian tubules of *Aedes albopictus* after a blood meal indicate an enhanced capacity for detoxification and excretion of metabolic wastes (20, 50). A similar enhanced capacity for detoxification and excretion of metabolic wastes is found here for *R. prolixus* when examining changes at 3 and 12 h PBM (**Figure 4**). Thus, the Malpighian tubule transcriptome in *R. prolixus* indicates that, as with other insects, *R. prolixus* Malpighian tubules have a high molecular capacity for all three phases of detoxification of xenobiotics and toxins.

### 4.1 ESTs

Most esterases (ESTs) belong to the CEs although other types of ESTs can be involved in different detoxification routes and may also play important roles in insecticide resistance (51). In detoxification, catalytic CEs are phase I enzymes responsible for hydrolyzing ester bonds, more specifically of carboxylic esters. *R. prolixus* Malpighian tubules express transcripts for several of the 22 members of the *R. prolixus* detoxification/dietary class of CEs (51) although they are not in high abundance (**Figure 4A**; **Supplementary Worksheets 1, 2**). Four of these transcripts are significantly upregulated at 3 h PBM (RPRC010228, RPRC007698, RPRC010265, and RPRC010268), while a different *CE* transcript (RPRC007696) is upregulated at 24 h PBM (**Figure 4A**). Of some curious interest in the class of CEs, however, is that a transcript for *acetylcholinesterase* is in high abundance and is upregulated significantly at 24 h PBM (**Figure 4A**; **Supplementary Worksheets 1, 2**). In a previous study (1) low levels of the transcript for the *muscarinic acetylcholine receptor* were reported expressed in the Malpighian tubule transcriptome. There is little to no understanding of the role of acetylcholine in peripheral tissues of insects.

### 4.2 CYP450s

The cytochrome P450 monooxygenases catalyze the conversion of a wide range of lipophilic compounds to more hydrophilic derivatives. Some of them play important roles in the metabolism of endogenous molecules and in degradation of xenobiotic compounds and are critical for phase II of detoxification (see 20, 51–53). The Malpighian tubules of unfed *Ae. albopicus* express 64 transcripts encoding putative CYP450s, with representatives from the mitochondrial, CYP2, CYP3, and CYP4 clades/clans (20). At each time point after a blood meal, several CYP450 transcripts from each clan are differentially expressed, with the exception of the CYP2 clan, and the changes are quite dynamic (20). The Malpighian tubules of *R. prolixus* express over 60 transcripts for putative CYP450s (**Supplementary Worksheets 1, 2**), with representatives from the mitochondrial, CYP2, CYP3, and CYP4 clans. In insects, members of the mitochondrial and CYP2 clans are involved with ecdysteroid production and developmental regulation (discussed later), whereas the CYP3 and CYP4 clans are typically associated with xenobiotic detoxification and insecticide resistance (52, 53). For review of these in *R. prolixus* and other hemipterans, see Volonté et al. (53). The *CYP2* clan is in quite low abundance in the *R. prolixus* Malpighian tubule transcriptome, followed by the *mitochondrial* clan and *CYP4* clan, but some members of the *CYP3* clan are in high abundance, suggesting potentially important detoxification roles of CYP3 members in the Malpighian tubules of *R. prolixus* (**Figure 4B**; **Supplementary Worksheets 1, 2**). As also described for *Ae. albopictus* (20) the *CYP3* clan reveals quite a dynamic pattern of differential expression PBM in *R. prolixus* tubules. In *R. prolixus*, at 3 h PBM, 9 transcripts are significantly downregulated with 3 upregulated, and at 24 h PBM, 17 transcripts are significantly downregulated with 8 upregulated (**Figure 4B**; **Supplementary Worksheets 1, 2**). In the *CYP4* clan, 1 transcript is downregulated and 3 are upregulated at 3 h PBM, and at 24 h PBM 3 are downregulated and 3 upregulated (**Figure 4B**; **Supplementary Worksheets 1, 2**). Clearly the response of *CYP3* and *CYP4* transcripts to blood-feeding is quite complex and experimentation is required to understand this process and the roles these clans may play in detoxification and pesticide resistance.

### 4.3 GSTs

GSTs also participate in phase II of detoxification and are present in most organisms, including hemipterans (51, 53). GSTs have been examined in the Malpighian tubules of *Ae. albopicus* (50). The cytosolic and microsomal GST transcripts are typically upregulated after blood meal, with at least 18 transcripts upregulated at each time point, and only 2-3 downregulated. Moreover, 14 transcripts are upregulated at 3 time points following the blood meal, in contrast to only one that is constitutively downregulated. Moreover, several GST transcripts that exhibited an upregulation at one or more time points after a blood meal have been implicated in metabolic resistance to insecticides in *Ae. aegypti* (see 20). Thus, the transcriptional regulation of GSTs appears to respond to a wide variety of detoxification and xenobiotic stresses.

In addition to the *CYP450s*, 12 transcripts encoding putative *GSTs* of the *Theta*, *Sigma*, *Omega*, *Zeta* and *microsomal* classes are present in the Malpighian tubule transcriptome of *R. prolixus* (**Figure 4C**; **Supplementary Worksheets 1, 2**), several of which are found in high abundance in tubules from unfed *R. prolixus*. Only 1 of 7 of the *Sigma* class is upregulated at 3 h PBM but 3 of 7 are significantly and highly upregulated at 24 h PBM (**Figure 4C**). Similarly, none of the *Theta* class are upregulated at 3 h PBM but 2 of 3 are upregulated at 24 h PBM. The *microsomal* class is significantly upregulated at 24 h PBM. Neither *Omega* nor *Zeta* transcripts are altered at 3 h PBM but both are downregulated at 24 h PBM. Thus, the transcriptional regulation of GSTs also appears to respond to blood gorging in *R. prolixus* in a dynamic manner.

### 4.4 ABC transporters

ABC transporters participate in phase III of detoxification (54) as multidrug transporters, reducing cellular concentrations of toxic xenobiotic compounds (see 55). In general, ABC transporters are promiscuous, transporting heme, glutathione-conjugated organic compounds, uric acid, xenobiotics, and lipids. In insects, the ABC protein family is divided into eight subfamilies, ABCA through ABCH (see 55). Members of the ABCB (Multidrug resistance, MDR), ABCC (MDR-associated proteins, MRPs), and ABCG subfamilies are implicated in cellular detoxification that contribute to resistance to toxins (see 55). Transcripts encoding ABC transporters are upregulated after a blood meal in *Ae. albopictus* (50), indicating an increase in Malpighian tubule capacity for ABC transporter-mediated detoxification after a blood meal (20, 50). Transcripts encoding all these putative ABC transporters are expressed in the *R. prolixus* Malpighian tubule transcriptome, some of which are of high abundance (**Figure 4D**; **Supplementary Worksheets 1, 2**). Similarly, these are enriched in the Malpighian tubules of *D. melanogaster* (56). In *R. prolixus* tubules, 8 transcripts are significantly upregulated and 10 downregulated at 3 h PBM, whereas 14 are upregulated and 12 downregulated at 24 h PBM (**Figure 4D**). Clearly, *R. prolixus* tubules have the capacity to engage in phase III of detoxification, but again, in a dynamic manner.

### 4.5 Heme and ROS

The utilization of blood as a source of nutrients constitutes an oxidative challenge to hematophagous insects. Blood processing in the digestive tract can result in the release of high concentrations of heme into the hemolymph, which may lead to iron-induced oxidative stress due to the generation of ROS.

To overcome the deleterious effects of heme, triatomines have developed several strategies to eliminate or neutralize heme. The first line of defense against heme toxicity in *R. prolixus* is heme polymerization into hemozoin in the digestive tract, which is then eliminated in the feces; an adaptation to hematophagy (57). A wide range of antioxidant enzymes also act in the digestive tract (58). Antioxidant enzyme machinery against ROS and cell detoxification, such as catalases, thioredoxin and glutathione peroxidases, CYP6X, along with some of the GSTs, show increased transcript levels in the Malpighian tubules at 24 h PBM reinforcing the importance of antioxidant enzymes in all tissues (**Figure 4E**; **Supplementary Worksheets 1, 2**). For example, the transcript for *glutathione peroxidase (GPx)* (RPRC011108), one of the major antioxidant proteins, is elevated at both 3 and 24 h PBM in Malpighian tubules in *R. prolixus*, and the transcripts for *catalase (Cat)* and for *thioredoxin peroxidase (TrX)* are abundant and significantly upregulated at 24 h PBM. In addition, the transcript of the antioxidant *xanthine dehydrogenase* (*XDH*) is highly abundant in tubules of *R. prolixus* and is significantly upregulated 24 h PBM (**Figure 4E**). Taken together, the presence of several peroxidase systems and antioxidant enzymes in Malpighian tubules indicates that the Malpighian tubules have the machinery to reduce the burden of ROS through different defense systems, maintaining the intracellular redox homeostasis in *R. prolixus*.

Another protective mechanism involves *R. prolixus* heme-binding protein (RHBP) which plays a protective role against heme-induced oxidative stress by inhibiting heme-induced lipid peroxidation and counteracting the deleterious effects of heme (59). Apo-RHBP circulates in the hemolymph and binds heme molecules that have crossed the midgut into the hemolymph. Once bound to RHBP, heme is no longer capable of catalyzing the generation of free radicals (59). RHBP is found in the fat body, but not in the flight muscles, anterior midgut, posterior midgut, or ovaries (60). More recently, however, RHBP was identified in the antenna of *R. prolixus* (61). Interestingly the *RHBP* transcript is abundantly expressed in *R. prolixus* Malpighian tubule transcriptome of unfed insects and is significantly upregulated at 3 h PBM, but not at 24 h PBM (**Figure 4E**). It would be interesting to monitor the expression of *RHBP* in the Malpighian tubules over a longer time course PBM since digestion of the blood meal only begins some days after ingestion in fifth instars.

These findings suggest that the Malpighian tubules of *R. prolixus* could contribute to antioxidant production and heme detoxification during blood meal processing, as is also proposed in mosquitoes (62).

### 4.6 Nitrogenous waste

In terrestrial insects, uric acid is the primary nitrogenous waste excreted (63). Uric acid, derived from amino acid and purine catabolism, can be excreted with minimal water, is relatively non-toxic, and may also serve as a valuable antioxidant (63, 64). In the Malpighian tubules of mosquito, transcriptomic and biochemical data suggest that the Malpighian tubules enhance their capacity to synthesize uric acid within 24 h post-blood meal (20) and likely play a key role in the excretion of uric acid following a blood meal. Also, in blood-fed mosquitoes, uric acid can be metabolized to urea and excreted through the uricolytic pathway. In *R. prolixus*, the uricolytic pathway has not been identified in the genome (28). Interestingly, as mentioned earlier, the transcript for *XDH* is upregulated 24 h PBM (**Figure 4E**), and XDH catalyzes the most downstream steps of uric acid synthesis (28). Thus, as with mosquitoes, *R. prolixus* Malpighian tubules may participate in uric acid synthesis. *R. prolixus* rapidly eliminates uric acid following blood gorging. Uric acid is transported into the lumen of the upper fluid secreting region of the Malpighian tubules, and then flows with the fluid into the lower tubule. Here, uric acid precipitates because of reabsorption of water and acidification (7). ABCG transporters have previously been suggested to transport uric acid (55), and interestingly, 2 *ABCG* transcripts (RPRC004060 and RPRC010941) are upregulated 24 h PBM (**Figure 4D**; **Supplementary Worksheets 1, 2**).

### 4.7 Calcium

*R. prolixus* copes with the large amounts of Ca^2+^ ingested in its blood meal by accumulating high concentrations of Ca^2+^ in the cells of the distal Malpighian tubules but not in the cells of the proximal Malpighian tubules; very little is actually excreted from the body (65). The Ca^2+^ accumulates in intracellular membrane-bound concretion bodies. Ca^2+^ deposited in these concretion bodies is also readily exchangeable, but the efflux preferentially passes to the hemolymph side of the tubule epithelium, and not into the lumen (hence it is not excreted). The *R. prolixus* Malpighian tubule transcriptome reveals numerous transcripts that encode for ion and water transport mechanisms, including transcripts for *Ca^2+^ channels* and transcripts for *potassium-dependent sodium-calcium exchangers* (*NCKX*) that could play critical roles in regulating intracellular Ca^2+^ levels (1). Thus, Malpighian tubules would appear to participate in Ca^2+^ homeostasis, but the mechanism of preferential accumulation and then release back into the hemolymph is not understood (see 1).

## 5 Other observations

### 5.1 Hormonal involvement

Hormones can enhance or suppress the immune response, thereby allowing the immune system to respond appropriately to changing internal states and external conditions (see 66). For example, reproduction and an immune defense are energetically demanding processes and a tradeoff between the two has been shown to occur in a diversity of female insects. Reproductive activity results in reduced immunity, and the converse, infection and thereby activation of the immune system, reduces reproductive output (67).

In *D. melanogaster*, and other insects, juvenile hormone (JH) can act as an antagonist of the immune response, and 20-hydroxyecdysone (20E) can potentiate immunity (40, 66). The Malpighian tubule transcriptome of *R. prolixus* reveals that the JH receptor *Methoprene tolerant* (*Met*) transcript is expressed in tubules from unfed insects, and is significantly upregulated at 3 h PBM, and downregulated at 24 h PBM (**Supplementary Figure 3A**). JH mediates one of its actions *via* the transcription factor Krüppel homolog 1 (Kr-h1), a key JH-early inducible gene. The transcript for *Kr-h1* is not present in the transcriptome of Malpighian tubules from unfed *R. prolixus* but is present, although in very small amounts, at 3 h PBM. Interestingly the transcript for *JH epoxide hydrolase* (*JHEH*) which codes for the enzyme that inactivates JH is very highly expressed in the tubule transcriptome and is upregulated 24 h PBM, suggesting that tubules may play a role in aspects of JH titer which are kept low in this penultimate instar (**Supplementary Figure 3A**). Curiously, transcripts for most of the enzymes in the biosynthetic pathway for JH are present in the tubule transcriptome (**Supplementary Worksheets 1, 2**); indeed, terpenoid biosynthesis has been identified by KEGG analysis as an enriched pathway occurring in Malpighian tubules after feeding (**Supplementary Figure 2**); however, there is no good evidence in insects that JH can be synthesized by tissues other than the corpora allata. Again, further research is required to investigate the possibility of JH production by Malpighian tubules.

In addition, the transcripts for the *ecdysone receptor* (*EcR*) and *ultraspiracle* (*USP*), which together form the 20E receptor complex, are also present, with *EcR* upregulated at 3 h and 24 h PBM, and *USP* upregulated at 3 h PBM (**Supplementary Figure 3B**). In addition, the ecdysone response genes transcripts (*E75, E74, Broad-complex (Br-C), FTZ-F, HR3*, and *HR4*) are present in the Malpighian tubule transcriptome and show variable up or downregulation at 3 h PBM but are stable at 24 h PBM. In addition, though, the transcript for *Shade* which codes for the enzyme that converts ecdysone (E) to 20E is also present and is significantly upregulated 3 h PBM (**Supplementary Figure 3B**). In this final instar before ecdysis into an adult, the 20E titer increases from a negligible level in the unfed insect to a detectable level within minutes following a blood meal (68). Thus, 20E is present at 3 h and 24 h PBM. These data certainly suggest that the *R. prolixus* Malpighian tubules not only can convert E to 20E, but also respond to 20E PBM. Of interest to this is the observation that ecdysteroids induce the expression of AMPs in *D. melanogaster* tubules with the different AMPs displaying differential expression in response to ecdysteroids. The early response gene *Br-C* appears to regulate the IMD pathway by activating *Relish* and physically interacting with it to activate AMPs expression (38). An early paper also suggested that Malpighian tubules of the tsetse fly *Glossina morsitans* respond to ecdysteroids with an increase in the rate of urine production, and that this might be one of the factors regulating ecdysteroid titer (69). Intriguingly, the Malpighian tubule transcriptome of *R. prolixus* also reveals transcripts for *Halloween genes* (**Supplementary Figure 3B**; **Supplementary Worksheets 1, 2**) that are involved in ecdysteroid biosynthesis (see 70). Some of these are in very low abundance, and so whether Malpighian tubules can synthesize ecdysteroids remains to be seen, but the possibility was also raised for Malpighian tubules of *Ae. albopictus* (20).

Several studies have examined the presence of transcripts for hormones and their receptors in insect Malpighian tubules, largely in the context of diuretic and antidiuretic hormones (1, 19–21). Similarly, a previous analysis of the *R. prolixus* transcriptome revealed the presence of G-protein coupled receptors for the known diuretic and antidiuretic hormones of *R. prolixus* (1). Interestingly, though, none of the transcripts for receptors for these diuretic hormones (serotonin, *R. prolixus* corticotropin-releasing factor diuretic hormone (Rhopr-CRF/DH) or the antidiuretic hormone *R. prolixus* cardioaccelatory peptide 2b (Rhopr-CAPA) are among the most abundant receptors expressed in the Malpighian tubule transcriptome. Of interest are GPCRs for ligands not previously tested for biological activity on the Malpighian tubules of *R. prolixus*. Indeed, the most abundant receptor transcript is that for the leucine-rich repeat-containing G protein-coupled receptor 1 (*LGR1*) for the glycoprotein hormone, GPA2/GPB5 (1). Another receptor transcript that is highly abundant is the *receptor-type guanylate cyclase* for the neuropeptide-like precursor peptide. Other previously unappreciated receptors that might be involved in Malpighian tubule function include orthologs of a neuroparsin receptor-like (Venus kinase receptor), parathyroid hormone-like receptor, adiponectin receptor, short neuropeptide F receptor (sNPFR), dopamine 1-like R2, ion transport peptide receptor, GABA receptor, the insulin receptor 1, and octopamine receptors (1). As discussed by Orchard et al. (1) these other receptors may be undescribed diuretic or antidiuretic hormones regulating post-prandial diuresis or may control some other functions of the Malpighian tubules such as those described in this review. For example, insulin-like peptides, octopamine, and sNPF have been shown to be involved in the immune response in a variety of insects (see 66), and so may also be involved in the immune response in *R. prolixus*.

Clearly there is the potential for hormonal control of Malpighian tubule immune response in these fifth instars, and it would be worthwhile investigating the Malpighian tubules from adult females to examine for reproduction/immune response tradeoffs.

### 5.2 Circadian pacemaker

The *D. melanogaster* circadian oscillator is well described and is composed of two intracellular feedback loops in gene expression: A *Period/Timeless* (*Per*/*Tim*) loop and a *Clock* (*Clk*) loop (see 71). Within these feedback loops, rhythmic transcription of clock genes is controlled *via* feedback from their own protein products; for example, the protein product PER is involved in a molecular feedback loop in which PER inhibits the transcription of its own mRNA. This feedback results in the PER protein cycling in a circadian manner, and this cycling in the brain is responsible for controlling a variety of circadian rhythms. PER has also been detected in non-neural tissues in the insect abdomen, including Malpighian tubules, and it has been suggested that Malpighian tubules contain a circadian pacemaker that functions independently of the brain (72). Here we find that many circadian rhythm gene transcripts are expressed in *R. prolixus* Malpighian tubules, including *Per*, *Tim*, and *Clk* (**Supplementary Figure 4**). The *Per* transcript is highly expressed in Malpighian tubules, and this, along with the transcripts for many clock genes is suggestive of tubules acting in a circadian manner. This matches results from mosquitoes wherein there appears to be a potential for rhythms in immunity, ROS detoxification, osmotic regulation, and metabolic detoxification (73). It should be noted here that for the current study in *R. prolixus*, although the *Per* transcript is downregulated at 24 h PBM, the insects were not necessarily dissected at the same time during the day/night cycle and so the experiment was not set up to monitor daily rhythms. Clearly though, this is an avenue of research to pursue.

PER protein stability is believed to be tightly regulated by phosphorylation (74, 75). Two *casein kinase 1* (*CK1*) genes, *CSNK1D* and *CSNK1E* which encode kinases (CK1δ and CK1ϵ, respectively), appear to regulate PER stability (76, 77). The mechanism by which these kinases control the clock is not known; however, PER is a direct target of all of these kinases, including casein kinase 2 (74, 78), and glycogen synthase kinase 3 (79). Here we note an increase in *CK1*δ and *CK1*ϵ transcripts at 24 h PBM in addition to all kinases involved in regulating PER stability (**Supplementary Figure 4**; **Supplementary Worksheets 1 and 2**), further supporting the notion that the Malpighian tubules express a circadian pacemaker that could function independently of the brain (72).

### 5.3 Energy budget

In invertebrates, it has generally been suggested that the energy demanded by diuresis is small when compared to the resting metabolism of the entire organism (80). However, due to the extraordinary ability to consume large amounts of blood in a single feed, *R. prolixus* diuresis represents an extreme case. It has been suggested that the production of urine by the Malpighian tubules in *R. prolixus* costs about 100 times more energy per unit time and unit weight than does the urine production in a typical mammalian kidney (80). Secretion by Malpighian tubules of *R. prolixus* fails in about 10-30 min without glucose, whereas with it, secretion persists for about 8-10 h (9). Clearly, Malpighian tubules are capable of utilizing carbohydrates to generate energy, presumably using the classical Glycolytic-Krebs cycle pathway. In fact, by KEGG pathways analysis, we can identify a number of DEG enriched at 3 h PBM that belong to the glycolysis signaling pathway (**Supplementary Figure 2**), where glucose is broken down in the cytosol in order to generate pyruvate molecules, which in turn are transformed into acetyl CoA in the mitochondria by the Krebs cycle. From here, the precursors produced will be used by oxidative phosphorylation to finally generate ATP. Most of the transcripts for the enzymes involved in these pathways are present in the Malpighian tubule transcriptome (**Supplementary Worksheets 1, 2**). Trehalose, a non-reducing disaccharide consisting of two glucose molecules, is the main carbohydrate found in insect hemolymph (81). A trehalose-specific facilitated transporter (TRET) leads to both the transfer of newly synthesized trehalose from the fat body into the circulating hemolymph and its subsequent uptake by other tissues. We recently identified the *TRET* transcript in *R. prolixus* (82) and reported its presence in the Malpighian tubules. Here, by transcriptome analysis, we find an increase of *TRET* transcript PBM as well as an upregulation of *membrane-bound trehalase*, responsible to convert trehalose into glucose to be used in cell metabolism, 3 *h* PBM concurrent with the energy demanding process of post-prandial diuresis (**Supplementary Worksheets 1, 2**). Thus, the glucose used for energy production in Malpighian tubules may be obtained from the hemolymph.

## 6 Final thoughts

*R. prolixus* Malpighian tubules have long been a model for post-prandial diuresis in insects (see 1), but an in-depth examination of their transcriptome shown here, indicates that they have the potential to participate in immune responses and detoxification; Malpighian tubules are much more than transporting epithelia for diuresis. Indeed, transcripts for many of the immune response and detoxification processes are expressed in the Malpighian tubules in unfed *R. prolixus* and are regulated 3 h PBM and 24 h PBM. This implies that *R. prolixus* tubules are “primed” to respond to challenges created by blood gorging; xenobiotics present in the blood or toxins produced by metabolism of the blood meal. Malpighian tubules clearly have the potential to neutralize and excrete such insults.

Although as described earlier, Malpighian tubules of other insects have been shown to be involved in stress, detoxification, and immunity (see 7, 16, 20, 22, 38, 50), their involvement in *R. prolixus* is not proven by this transcriptome analysis. It will be necessary to validate the transcript expression using qPCR, to monitor their translation into protein, and to challenge *R. prolixus* with xenobiotics to monitor the response of Malpighian tubules. Indeed, the *R. prolixus* used here were merely provided with a blood meal and examined at 3 h and 24 h PBM, but digestion in fifth instars does not begin until some days after feeding and the blood used for this feeding was aseptic! So, along with further analysis on transcripts that may be missing from the various pathways, is the need to interrogate the tubules over a longer time course PBM, and under controlled challenges (xenobiotics, toxins, etc.).

The transcriptome also highlights the possible hormonal control over Malpighian tubules, and/or involvement of tubules in regulating hormonal titers, including those of JH and ecdysteroids, and a variety of neuropeptides, as has been suggested for tubules of *D. melanogaster* and *Ae*. *albopictus* (20, 50, 66, 67).

Finally, identifying transcripts for genes associated with circadian rhythm suggests a whole other mechanism of control over tubules. Such rhythms have been suggested in tubules of *D. melanogaster* (72) and in mosquitoes, rhythms associated with processes such as immunity, detoxification, and osmotic regulation (see 73) have been described, but this area clearly needs more attention.

Overall, this analysis leads to new experimentation and the opening of *R. prolixus* as a model for Malpighian tubules quite separate from diuresis alone; to an appreciation of the central importance that the tubules might play in immunity and detoxification, how these may be influenced by hormones and their second messengers, and how their responses may be influenced by circadian clocks. As has been pointed out before (40), the importance of Malpighian tubules lies in the fact that they are essentially free floating in the hemolymph and perhaps one of the first epithelial tissues to sense and respond to pathogens and toxins that have invaded/appeared in, the hemolymph. Transcriptomics has further emphasized that there is much more to be learned and much that we do not understand about *R. prolixus* Malpighian tubules. Most importantly, though, transcriptomics has identified novel avenues for future research.

## Author contributions

IO, AA-D, JL and AL contributed to conception and design of the study. AA-D and JL organized the database and statistical analysis. IO wrote the first draft of the manuscript. IO, AA-D, JL, AL wrote sections of the manuscript. All authors contributed to the article and approved the submitted version.

## References

[1] Orchard I Leyria J Al-Dailami A Lange AB. Fluid secretion by Malpighian tubules of *Rhodnius prolixus*: Neuroendocrine control with new insights from a transcriptome analysis. Front Endocrinol (2021) 12:72248. doi: 10.3389/fendo.2021.72248 PMC8426621 34512553

[2] Lange AB Leyria J Orchard I. The hormonal and neural control of egg production in the historically important model insect, *Rhodnius prolixus*: A review, with new insights in this post-genomic era. Gen Comp Endocrinol (2022) 321-322:114030. doi: 10.1016/j.ygcen.2022.114030 35317995

[3] Wigglesworth VB. Factors controlling moulting and ‘metamorphosis’ in an insect. Nature (1934) 133:725–6. doi: 10.1038/133725b0

[4] Wigglesworth VB. The principles of insect physiology. New York: Chapman and Hall (1972). 827 p.

[5] Edwards JS. Sir Vincent wigglesworth and the coming of age of insect development. Int J Dev Biol (1998) 42:471–3. 9654033

[6] Davey KG. The interaction of feeding and mating in the hormonal control of egg production in *Rhodnius prolixus* . J Insect Physiol (2007) 53:208–15. doi: 10.1016/j.jinsphys.2006.10.002 17126364

[7] O’Donnell MJ. Too much of a good thing: how insects cope with excess ions or toxins in the diet. J Exp Biol (2009) 212:363–72. doi: 10.1242/jeb.023739 19151211

[8] Maddrell SHP. Functional design of the neurosecretory system controlling diuresis in *Rhodnius prolixus* . Amer Zool (1976) 16:131–9. doi: 10.1093/icb/16.2.131

[9] Maddrell SHP. Secretion by the Malpighian tubules of *Rhodnius*. the movements of ions and water. J Exp Biol (1969) 51:71–97. doi: 10.1242/jeb.51.1.71

[10] Maddrell SHP. A diuretic hormone in *Rhodnius prolixus* Stål. Nature (1962) 194:605–6. doi: 10.1038/194605b0

[11] Maddrell SHP. The site of release of the diuretic hormone in *Rhodnius prolixus* - a new neurohaemal system in insects. J Exp Biol (1966) 45:499–508. doi: 10.1242/jeb.45.3.499

[12] Maddrell SHP Herman WS Farndale JA Riegel JA. Synergism of hormones controlling epithelial fluid transport in an insect. J Exp Biol (1993) 174:65–80. doi: 10.1242/jeb.174.1.65

[13] Blumenthal E. Regulation of chloride permeability by endogenously produced tyramine in the *Drosophila* Malpighian tubule. Am J Physiol Cell Physiol (2003) 284:C718–28. doi: 10.1152/ajpcell.00359.2002 12444020

[14] Beyenbach KW. Transport mechanisms of diuresis in Malpighian tubules of insects. J Exp Biol (2003) 206:3845–56. doi: 10.1242/jeb.00639 14506220

[15] Beyenbach KW Skaer H Dow JAT. The developmental, molecular, and transport biology of Malpighian tubules. Annu Rev Entomol (2010) 55:351–74. doi: 10.1146/annurev-ento-112408-085512 19961332

[16] Dow JAT Davies SA. The Malpighian tubule: Rapid insights from post-genomic biology. J Insect Physiol (2006) 52:365–78. doi: 10.1016/j.jinsphys.2005.10.007 16310213

[17] Dow JAT. Insights into the Malpighian tubule from functional genomics. J Exp Biol (2009) 212:435–45. doi: 10.1242/jeb.024224 19151219

[18] Dow JAT Pandit A Davies SA. New views on the Malpighian tubule from post-genomic technologies. Curr Opin Insect Sci (2018) 29:7–11. doi: 10.1016/j.cois.2018.05.010 30551828

[19] Overend G Cabrero P Halberg KA Ranford-Cartwright LC Woods DJ Davies SA. A comprehensive transcriptomic view of renal function in the malaria vector, *Anopheles gambiae* . Insect Biochem Mol Biol (2015) 67:47–58. doi: 10.1016/j.ibmb.2015.05.007 26003916

[20] Esquivel SJ Cassone BJ Piermarini PM. A *de novo* transcriptome of the Malpighian tubules in non-blood-fed and blood-fed Asian tiger mosquitoes *Aedes albopictus*: Insights into diuresis, detoxification, and blood meal processing. PeerJ (2016) 4:e1784. doi: 10.7717/peerj.1784 26989622 PMC4793337

[21] Xu J Liu Y Li H Tarashansky AJ Kalicki CH Hung R-J. Transcriptional and functional motifs defining renal function revealed by single-nucleus RNA sequencing. PNAS (2022) 119:e2203179119. doi: 10.1073/pnas.2203179119 35696569 PMC9231607

[22] Sneed SD Dwivedi SB DiGate C Denecke S Povelones M. *Aedes aegypti* Malpighian tubules are immunologically activated following systemic toll activations. Parasites Vectors (2022) 15:469. doi: 10.1186/s13071-022-05567-2 36522779 PMC9753289

[23] Ligoxygakis P. “Insect immunity”. In: Ligoxygakis P, editor. Advances in insect physiology, vol. 52 . Cambridge, MA: Academic Press (2017). p. 1–248.

[24] Salcedo-Porras N Lowenberger C. “The immune system of triatomines”. In: Guareri A Lorenzo M, editors. Triatominae – the biology of chagas disease vectors. Switzerland: Springer Nature Switzerland AG (2021). p. P. 307–344.

[25] Zeng T Jaffar S Xu Y Qi Y. The intestinal immune defense system in insects. Intern J Molec Sci (2022) 23:15132. doi: 10.3390/ijms232315132 PMC9740067 36499457

[26] Farina P Bedini S Conti B. Multiple functions of Malpighian tubules in insects: A review. Insects (2022) 13:1001. doi: 10.3390/insects13111001 36354824 PMC9697091

[27] Salcedo-Porras N Guarneri A Oliveira P Lowenberger C. *Rhodnius prolixus*: Identification of missing components of the IMD immune signaling pathway and functional characterization of its role in eliminating bacteria. PloS One (2019) 12:e0214794. doi: 10.1371/journal.pone.0214794 PMC6447187 30943246

[28] Mesquita RD Vionette-Amaral RJ Lowenberger C Rivera-Pomar R Monteiro FA Minx P. Genome of *Rhodnius prolixus*, an insect vector of chagas disease, reveals unique adaptations to hematophagy and parasite infection. Proc Natl Acad Sci USA (2015) 112:14936–41. doi: 10.1073/pnas.1506226112 PMC4672799 26627243

[29] Leyria J Orchard I Lange AB. Transcriptomic analysis of regulatory pathways involved in female reproductive physiology of *Rhodnius prolixus* under different nutritional states. Sci Rep (2020) 10:11431. doi: 10.1038/s41598-020-67932-4 32651410 PMC7351778

[30] Leyria J Orchard I Lange AB. What happens after a blood meal? a transcriptome analysis of the main tissues involved in egg production in *Rhodnius prolixus*, an insect vector of chagas disease. PloS Negl Trop Dis (2020) 14:e0008516. doi: 10.1371/journal.pntd.0008516 33057354 PMC7591069

[31] Kimbrell D Beutler B. The evolution and genetics of innate immunity. Nat Rev Genet (2001) 2:256–67. doi: 10.1038/35066006 11283698

[32] Tanji T Hu X Weber ANR Ip T. Toll and IMD pathways synergistically activate an innate immune response in *Drosophila melanogaster* . Mol Cell Biol (2007) 27:12. doi: 10.1128/MCB.01814-06 PMC1900069 17438142

[33] Hoffmann J. The immune response of *Drosophila* . Nature (2003) 426:33–8. doi: 10.1038/nature02021 14603309

[34] Mondotte JA Saleh M-C. “Antiviral immune response and the route of infection in *Drosophila melanogaster* .” In: Kielian M Mettenleiter TC Roossinck MJ, editors. Advances in virus research, vol. 100 . Academic Press (2018). p. 247–78. doi: 10.1016/bs.aivir.2017.10.006 29551139

[35] Lemaitre B Hoffmann J. The host defense of *Drosophila melanogaster* . Annu Rev Immunol (2007) 25:697–743. doi: 10.1146/annurev.immunol.25.022106.141615 17201680

[36] Vieira CS Mattos DP Waniek PJ Santangelo JM Figueiredo MB Gumiel M. *Rhodnius prolixus* interaction with *Trypanosoma rangeli*: modulation of the immune system and microbiota population. Parasites Vectors (2015) 8:135. doi: 10.1186/s13071-015-0736-2 25888720 PMC4350287

[37] Vieira CS Waniek PJ Castro DP Mattos DP Moreira OC Azambuja P. Impact of *Trypanosoma cruzi* on antimicrobial peptide gene expression and activity in the fat body and midgut of *Rhodnius prolixus* . Parasites Vectors (2016) 9:119. doi: 10.1186/s13071-016-1398-4 26931761 PMC4774030

[38] Verma P Tapadia MG. Early gene broad complex plays a key role in regulating the immune response triggered by ecdysone in the Malpighian tubules of *Drosophila melanogaster* . Mol Immunol (2015) 66:325–39. doi: 10.1016/j.molimm.2015.03.249 25931442

[39] Davies S-A Overend G Sebastian S Cundall M Cabrero P Dow JAT. Immune and stress response ‘cross-talk’ in the *Drosophila* Malpighian tubule. J Insect Physiol (2012) 58:488–97. doi: 10.1016/j.jinsphys.2012.01.008 22306292

[40] Tapadia MG Verma P. Immune response and anti-microbial peptides expression in Malpighian tubules of *Drosophila melanogaster* is under developmental regulation. PloS One (2012) 7:e40714. doi: 10.1371/journal.pone.0040714 22808242 PMC3395640

[41] Yuan F Wei C. Gene expression profiles in Malpighian tubules of the vector leafhopper *Psammotettix striatus* (L.) revealed regional functional diversity and heterogeneity. BMC Genom (2022) 23:67. doi: 10.1186/s12864-022-08300-6 PMC8781387 35057738

[42] Souza-Neto JA Sim S Dimopoulos G. An evolutionary conserved function of the JAK-STAT pathway in anti-dengue defense. PNAS (2009) 106:17841–6. doi: 10.1073/pnas.0905006106 PMC2764916 19805194

[43] Ursic-Bedoya R Buchhop J Joy JB Durvasula R Lowenberger C. Prolixicin: A novel antimicrobial peptide isolated from *Rhodnius prolixus* with differential activity against bacteria and *Trypanosoma cruzi* . Insect Mol Biol (2011) 20:775–86. doi: 10.1111/j.1365-2583.2011.01107.x 21906194

[44] Wang X Luo H Zhang R. Innate immune response in the Chinese oak silkworm, *Antheraea pernyi* . Dev Comp Immunol (2018) 83:22–33. doi: 10.1016/j.dci.2017.12.010 29241953

[45] Mulnix AB Dunn PE. Structure and induction of a lysozyme gene from the tobacco hornworm, *Manduca sexta* . Insect Biochem Molec Biol (1994) 24:271–81. doi: 10.1016/0965-1748(94)90007-8 7517269

[46] Ursic-Bedoya RJ Nazzari H Cooper D Triana O Wolff M Lowenberger C. Identification and characterization of two novel lysozymes from *Rhodnius prolixus*, a vector of chagas disease. J Insect Physiol (2008) 54:593–603. doi: 10.1016/j.jinsphys.2007.12.009 18258253

[47] Zhao P Li J Wang Y Jiang H. Broad-spectrum antimicrobial activity of the reactive compounds generated *in vitro* by *Manduca sexta* phenoloxidase. Insect Biochem Mol Biol (2007) 37:952–9. doi: 10.1016/j.ibmb.2007.05.001 PMC2047599 17681234

[48] Kim S-H Lee W-J. Role of DUOX in gut inflammation: Lessons from *Drosophila* model of gut-microbiota interactions. Front Cell Infect Microbiol (2014) 3:116. doi: 10.3389/fcimb.2013.00116 24455491 PMC3887270

[49] Wang X-H Aliyari R Li W-X Kim K Carthew R Atkinson P. RNA Interference directs innate immunity against viruses in adult *Drosophila* . Science (2006) 312:452–4. doi: 10.1126/science.1125694 PMC1509097 16556799

[50] Esquivel CJ Cassone BJ Piermarini PM. Transcriptomic evidence for a dramatic functional transition of the Malpighian tubules after a blood meal in the Asian tiger mosquito *Aedes albopictus* . PloS Negl Trop Dis (2014) 8:e2929. doi: 10.1371/journal.pntd.0002929 24901705 PMC4046972

[51] Schama R Pedrini N Juárez MP Nelson DR Torres AQ Valle D. *Rhodnius prolixus* supergene families of enzymes potentially associated with insecticide resistance. Insect Biochem Mol Biol (2016) 69:91–104. doi: 10.1016/j.ibmb.2015.06.005 26079630

[52] Feyereisen R. Evolution of insect P450. Biochem Soc Trans (2006) 34:1252–5. doi: 10.1042/BST0341252 17073796

[53] Volonté M Traverso L Estivalis JML Almeida FC Ons S. Comparative analysis of detoxification-related gene superfamilies across five hemipteran species. BMC Genomics (2022) 23:757. doi: 10.1186/s12864-022-08974-y 36396986 PMC9670383

[54] Lage H. ABC-Transporters: implications on drug resistance from microorganisms to human cancers. Int J Antimicrob Agents (2003) 22:188–99. doi: 10.1016/S0924-8579(03)00203-6 13678820

[55] Dermauw W Van Leeuwen T. The ABC gene family in arthropods: Comparative genomics and role in insecticide transport and resistance. Insect Biochem Mol Biol (2014) 45:89–110. doi: 10.1016/j.ibmb.2013.11.001 24291285

[56] Robinson SW Herzyk P Dow JAT Leader DP. FlyAtlas: database of gene expression in the tissues of *Drosophila melanogaster* . Nucleic Acids Res (2013) 41:D744–750. doi: 10.1093/nar/gks1141 PMC3531048 23203866

[57] Oliveira MF Silva JR Dansa-Petretski M de Souza W Braga CMS Masuda H. Haemozoin formation in the midgut of the blood-sucking insect *Rhodnius prolixus* . FEBS Lett (2000) 477:95–8. doi: 10.1016/S0014-5793(00)01786-5 10899317

[58] Paiva-Silva GO Cruz-Oliveira C Nakayasu ES Maya-Monteiro CM Dunkov BC Masuda H. A heme-degradation pathway in a blood-sucking insect. PNAS (2006) 102:8030–5. doi: 10.1073/pnas.0602224103 PMC1472424 16698925

[59] Dansa-Petretski M Ribeira JMC Atella GC Masuda H Oliveira PL. Antioxidant role of *Rhodnius prolixus* heme-binding protein, protection against heme-induced lipid peroxidation. J Biol Chem (1995) 270:10893–6. doi: 10.1074/jbc.270.18.10893 7738029

[60] Paiva-Silva GO Sorgine MHF Benedetti CE Meneghini R Almeida IC Machado EA. On the biosynthesis of *Rhodnius prolixus* heme-binding protein. Insect Biochem Mol Biol (2002) 32:1533–41. doi: 10.1016/S0965-1748(02)00074-7 12530221

[61] Oliveira DS Brito NF Franco TA Moreira MF Leal WS Melo ACA. Functional characterization of odorant binding protein 27 (RproOBP27) from *Rhodnius prolixus* antennae. Front Physiol (2018) 9:1175. doi: 10.3389/fphys.2018.01175 30210359 PMC6119777

[62] Piermarini PM Esquivel CJ Denton JS. Malpighian tubules as novel targets for mosquito control. Int J Environ Res Public Health (2017) 14:111. doi: 10.3390/ijerph14020111 28125032 PMC5334665

[63] Dow AT. The versatile stellate cell – more than just a space-filler. J Insect Physiol (2012) 58:467–72. doi: 10.1016/j.jinsphys.2011.12.003 22202730

[64] Ramsey JS MacDonald SJ Jander G Nakabachi A Thomas GH Douglas AE. Genomic evidence for complementary purine metabolism in the pea aphid, *Acyrthosiphon pisum*, and its symbiotic bacterium buchnera aphidicola. Insect Mol Biol (2010) 19:241–8. doi: 10.1111/j.1365-2583.2009.00945.x 20482654

[65] Maddrell SHP Whittembury G Mooney RL Harrison JB Overton JA Rodriquez B. The fate of calcium in the diet of *Rhodnius prolixus*: Storage in concretion bodies in the Malpighian tubules. J Exp Biol (1991) 157:483–502. doi: 10.1242/jeb.157.1.483 2061707

[66] Nunes C Sucena E Koyama T. Endocrine regulation of immunity in insects. FEBS J (2021) 288:3928–47. doi: 10.1111/febs.15581 33021015

[67] Schwenke RA Lazzaro BP Wolfner MF. Reproduction – immunity trade-offs in insects. Annu Rev Entomol (2016) 61:239–56. doi: 10.1146/annurev-ento-010715-023924 PMC5231921 26667271

[68] Steel CGH Bollenbacher WE Smith SL Gilbert LI. Haemolymph ecdysteroid titres during larval-adult development in *Rhodnius prolixus*: Correlations with moulting hormone action and brain neurosecretory cell activity. J Insect Physiol (1982) 28:519–25. doi: 10.1016/0022-1910(82)90032-4

[69] Gee JD Whitehead DL Koolman J. Steroids stimulate secretion by insect Malpighian tubules. Nature (1977) 269:238–9. doi: 10.1038/269238a0

[70] Benrabaa SAM Orchard I Lange AB. The role of ecdysteroid in the regulation of ovarian growth and oocyte maturation in *Rhodnius prolixus*, a vector of chagas disease. J Exp Biol (2022) 225:jeb244830. doi: 10.1242/jeb.244830 36268612

[71] Hardin PE. The circadian timekeeping system of *Drosophila* . Curr Biol (2005) 15:714–22. doi: 10.1016/j.cub.2005.08.019 16139204

[72] Hege DM Stanewsky R Hall JC Giebultowica JM. Rhythmic expression of a PER-reporter in the Malpighian tubules of decapitated *Drosophila:* evidence for a brain-independent circadian clock. J Biol Rhythms (1997) 12:300–8. doi: 10.1177/074873049701200402 9438878

[73] Rund SSC O’Donnell AJ Gentile JE Reece SE. Daily rhythms in mosquitoes and their consequence for malaria transmission. Insects (2016) 7:14. doi: 10.3390/insects7020014 27089370 PMC4931426

[74] Edery I Zwiebel LJ Dembinska ME Rosbash M. Temporal phosphorylation of the *Drosophila* period protein. Proc Natl Acad Sci USA (1994) 91:2260–4. doi: 10.1073/pnas.91.6.2260 PMC43350 8134384

[75] Lee C Etchegaray JP Cagampang FR Loudon A Reppert SM. Posttranslational mechanisms regulate the mammalian circadian clock. Cell (2001) 107:855–67. doi: 10.1016/S0092-8674(01)00610-9 11779462

[76] Zhou M Kim JK Eng GWL Forger DB Virshup DM. A Period2 phosphoswitch regulates and temperature compensates circadian period. Mol Cell (2015) 60:77–88. doi: 10.1016/j.molcel.2015.08.022 26431025

[77] Eide EJ Woolf MF Kang H Woolf P Hurst W Camacho F. Control of mammalian circadian rhythm by CKIϵ-regulated proteasome-mediated PER2 degradation. Mol Cell Biol (2005) 25:2795–807. doi: 10.1128/MCB.25.7.2795-2807.2005 PMC1061645 15767683

[78] Aryal RP Kwak PB Tamayo AG Gebert M Chiu P-L Walz T. Macromolecular assemblies of the mammalian circadian clock. Mol Cell (2017) 67:770–782.e6. doi: 10.1016/j.molcel.2017.07.017 28886335 PMC5679067

[79] Fustin J-M Kojima R Itoh K Okamura H. Two Ck1δ transcripts regulated by m6A methylation code for two antagonistic kinases in the control of the circadian clock. Proc Natl Acad Sci USA (2018) 115:5980–5. doi: 10.1073/pnas.1721371115 PMC6003373 29784786

[80] Florey E. Excretion in insects: Energetics and functional principles. J Exp Biol (1982) 99:417–24. doi: 10.1242/jeb.99.1.417

[81] Elbein AD Pan YT Pastuszak I Carroll D. New insights on trehalose: a multifunctional molecule. Glycobiology (2003) 13:17R–27R. doi: 10.1093/glycob/cwg047 12626396

[82] Leyria J El-Mawed H Orchard I Lange AB. Regulation of a trehalose-specific facilitated transporter (TRET) by insulin and adipokinetic hormone in *Rhodnius prolixus*, a vector of chagas disease. Front Physiol (2021) 12:624165. doi: 10.3389/fphys.2021.624165 33643069 PMC7902789

