# Supplementary material for: Malpighian tubules of Rhodnius prolixus: More than post-prandial diuresis
Source: Front Insect Sci. 2023 Mar 30;3:1167889. doi: 10.3389/finsc.2023.1167889 (PMC10926411; doi:10.3389/finsc.2023.1167889)
Supplement: Supplementary file 1 [file DataSheet_1.docx]

**Malpighian tubules of *Rhodnius prolixus*: More than post-prandial diuresis**

Ian Orchard, Areej Al-Dailami, Jimena Leyria and Angela B. Lange

Department of Biology, University of Toronto Mississauga, Mississauga, ON, Canada

* Corresponding author: Ian Orchard: [ian.orchard@utoronto.ca](mailto:ian.orchard@utoronto.ca)

Supplementary Figures 1-4

**
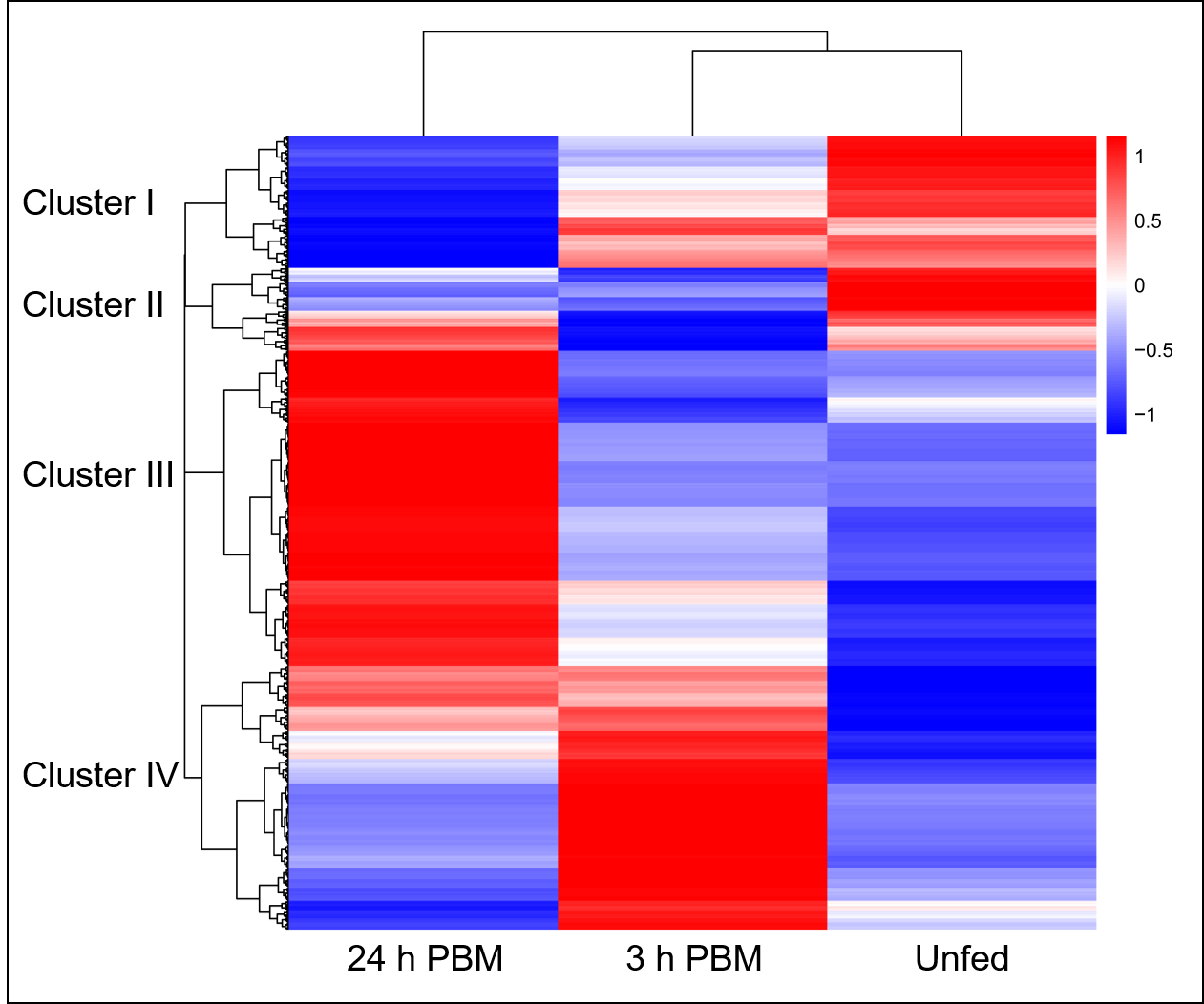
**

**Supplementary Figure 1: Heat map cluster of the differentially expressed genes that are regulated by the nutritional condition.** The log_10_(FPKM+1) for each gene was used for hierarchical analysis of heat map at each of the 3 nutritional condition: unfed, 3 h PBM and 24 h PBM. Red denotes genes with high expression levels, and blue denotes genes with low expression levels. The color ranging from red to blue indicates values from large to small. The vertical distances on each branch of the dendrogram represent the degree of similarity between gene expression profiles of the different samples. h PBM, hours post blood meal.

**
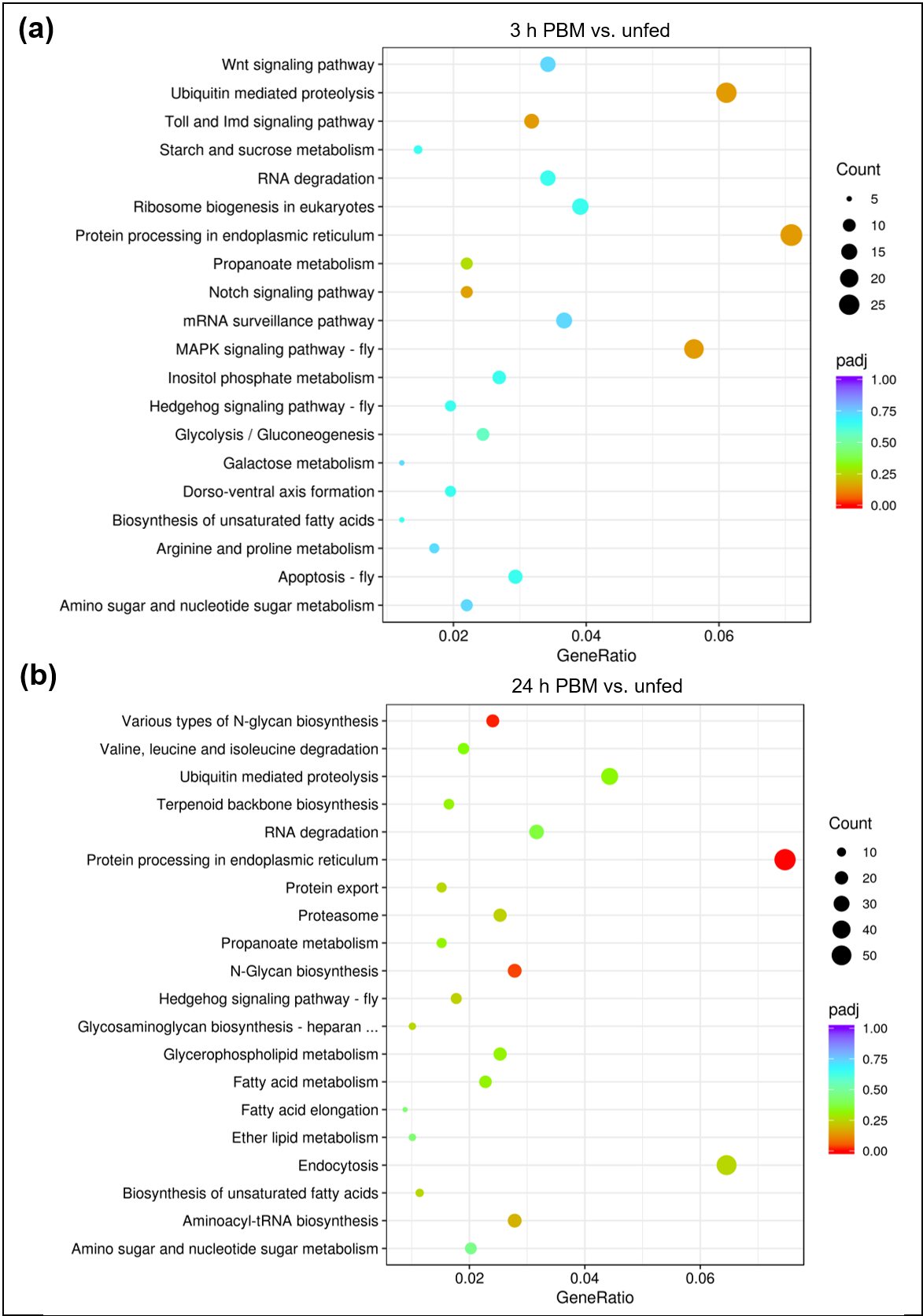
**

**Supplementary Figure 2: Scatter plots of the main KEGG pathways analyzed from the transcriptome of the Malpighian tubules at different nutritional conditions.** **(a-b)** Dot size indicates k/n ratio (*gene ratio*), where k is the number of genes participating in the current KEGG pathway and n is the number of genes annotated as participants of any KEGG pathway. Dot color indicates the enrichment test FDR (Fisher's exact test). Highlighted KEGG terms are shown with arrows. The DEGs were mapped to the KEGG terms in the database (<https://www.genome.jp/kegg/>). padj, p-value adjusted; h PBM, hours post blood meal.


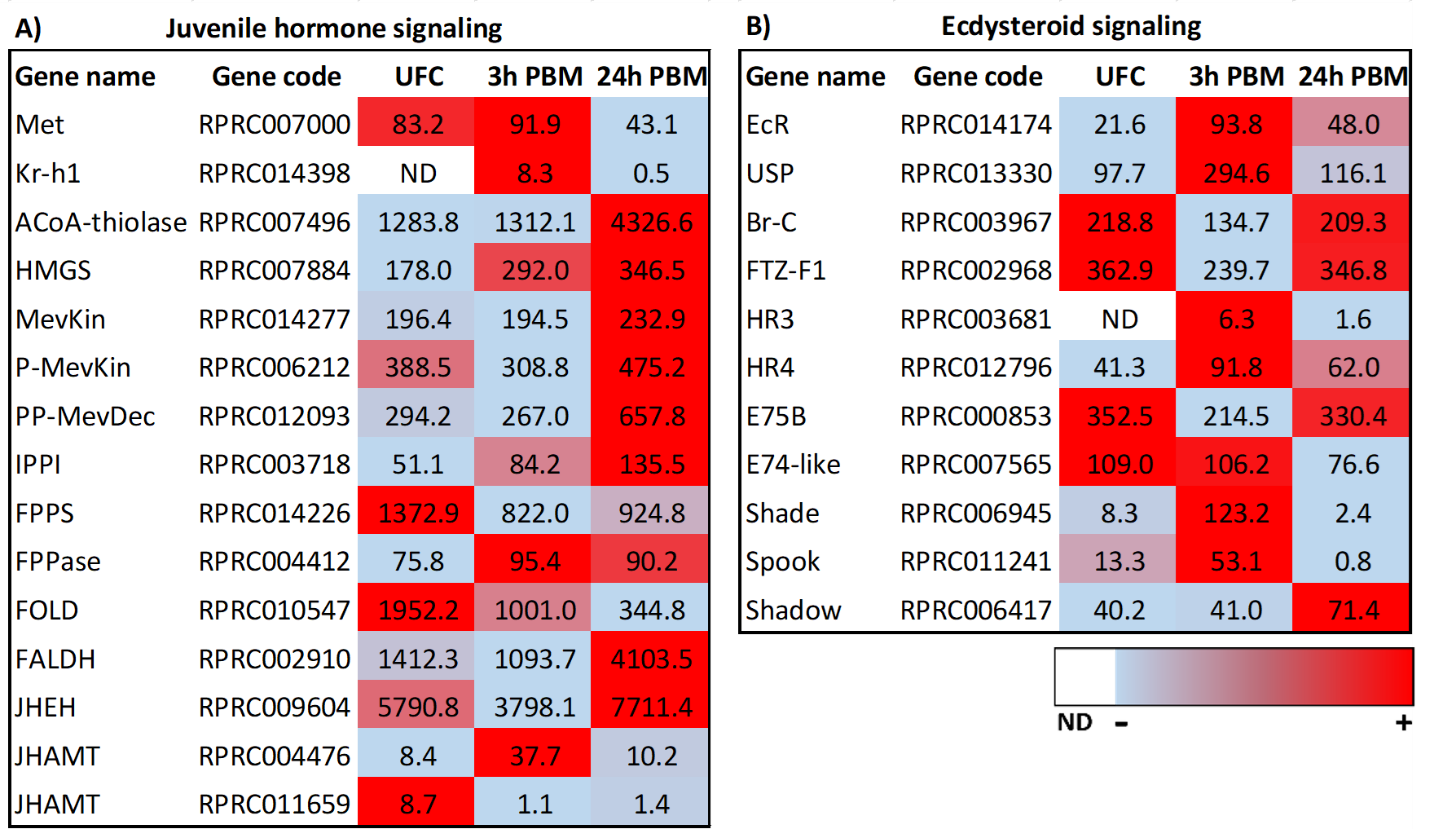


**Supplementary Figure 3.** Heat map comparing the mRNA expression levels of **(A)** Juvenile hormone signaling and **(B) Ecdysteroid signaling** in Malpighian tubules from insects in the unfed condition (UFC), 3 h and 24 h post blood meal (PBM). The input data is the readcount value from gene expression level analysis after normalization and is presented by means of a color scale, in which light blue/red represent lowest/highest expression and white indicate transcript expression is 0 or not detected (ND). DESeq was used to perform the analysis. Details of transcript expression are shown in Supplementary Worksheets 1 and 2.


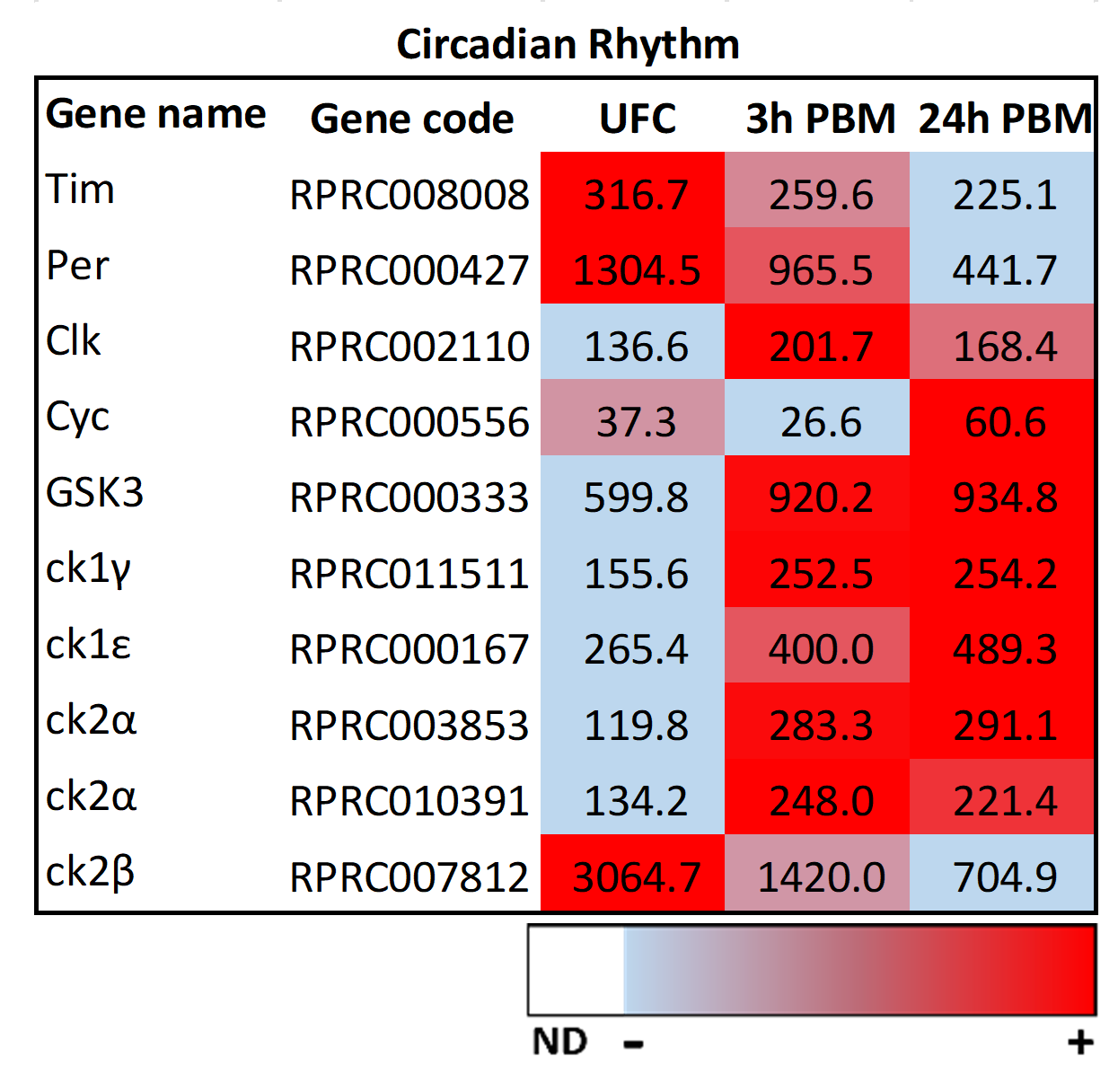


**Supplementary Figure 4.** Heat map comparing the mRNA expression levels of circadian rhythm genes in Malpighian tubules from insects in the unfed condition (UFC), 3 h and 24 h post blood meal (PBM). The input data is the readcount value from gene expression level analysis after normalization and is presented by means of a color scale, in which light blue/red represent lowest/highest expression and white indicate transcript expression is 0 or not detected (ND). DESeq was used to perform the analysis. Details of transcript expression are shown in Supplementary Worksheets 1 and 2.
